# Assessing the effects of hybridization and precipitation on invasive weed demography using strength of selection on vital rates

**DOI:** 10.1186/s12862-016-0833-7

**Published:** 2016-12-07

**Authors:** Zachary Teitel, Agnieszka Klimowski, Lesley G. Campbell

**Affiliations:** 1 Department of Chemistry and Biology, Ryerson University, 350 Victoria Street, Toronto, ON M5B 2K3 Canada; 2 Current Address: Department of Integrative Biology, University of Guelph, 50 Stone Road East, Guelph, ON N1G 2W1 Canada; 3 Current Address: Department of Physical and Environmental Sciences, University of Toronto Scarborough, 1265 Military Trail, Toronto, ON M1C 1A4 Canada

**Keywords:** (3-10): Demography, Experimental evolution, Gene flow, Global climate change, Life Table Response Experiment, *Raphanus raphanistrum*, *Raphanus sativus*, Rain-out shelter

## Abstract

**Background:**

As global climate change transforms average temperature and rainfall, species distributions may meet, increasing the potential for hybridization and altering individual fitness and population growth. Altered rainfall specifically may shift the strength and direction of selection, also manipulating population trajectories. Here, we investigated the role of interspecific hybridization and selection imposed by rainfall on the evolution of weedy life-history in non-hybrid (*Raphanus raphanistrum*) and hybrid (*R. raphanistrum x R. sativus*) populations using a life table response experiment.

**Results:**

In documenting long-term population dynamics, we determined intrinsic (*r*) and asymptotic (*λ*) population growth rates and sensitivities, a measure of selection imposed on demographic rates. Hybrid populations experienced 8.7-10.3 times stronger selection than wild populations for increased seedling survival. Whereas crop populations generally exhibit little dormancy and wild populations often exhibit dormancy, non-hybrid populations experienced 10% stronger selection than hybrid populations for exhibiting seed dormancy. Selection on survival-to-flowering in wild, not hybrid, populations declined marginally with increasing soil moisture. Hybrid populations exhibited greater *r*, but not *λ*, than wild populations regardless of moisture environment. In general, fecundity contributed most to differences in λ but fecundity only contributed positively to hybrid λ relative to wild λ when precipitation was altered (either higher or lower than control) and not under control watering conditions.

**Conclusions:**

Selection on key demographic traits may not change dramatically in response to rainfall, and hybridization may more strongly influence the demography of these weedy species than rainfall. If hybrid populations can respond to selection for increased dormancy, this may make it more difficult to deplete weed seed banks and increase the persistence of crop genes in weed populations.

## Background

Hybridization between crops and their wild and weedy relatives instantaneously changes the genetic composition of weed populations [1] and thus may influence phenotypic evolution and success of weedy or invasive populations [2–4]. Crop-wild hybridization may result in either the transfer of adaptive, crop alleles to weed populations [5] or the generation of unique hybrid phenotypes via transgressive segregation [6]. In either case, crop-wild hybrid weed populations may exhibit significantly different phenotypes relative to ancestral wild and/or weedy populations [7–9]. The fitness advantage of these new weedy phenotypes is often context dependent [10, 11]. For instance, under stressful agricultural conditions that accompany the application of herbicides or weed-competition gradients, sometimes weed fitness can be enhanced by the acquisition of crop traits. In contrast, under less selective environments, the acquisition of crop genes may reduce weed fitness [12, 13]. Furthermore, traits that are beneficial for weeds in agricultural contexts can vary regionally due to the arrangement of genetic variation in geographic selection mosaics [14]. Therefore, evaluating the ecological consequences of crop-wild hybridization in one environmental context (vs. many) may under- or overestimate the potential success of hybrid weed populations [3, 15].

If fitness advantages of advanced-generation hybrids are context-dependent, then one might predict that environmental gradients should be important influences on population demography [16]. For instance, under some stressful conditions (e.g., herbicide application or competition), weed fitness can be boosted by acquiring crop traits [12, 13]. Where broad environmental clines are important for defining population dynamics, changes to these climatic conditions may alter the magnitude and direction of selection acting on plants [17] and thus impact population growth. However, selection changing with environmental gradients is context-dependent [18, 19], and population dynamics are also influenced by the relative frequency of genotypes within that population [20, 21].

Providing that abiotic selection pressures vary across environmental clines [19, 22], the persistence of crop-wild hybrid populations may be determined by the strength of selection acting on fitness-correlated traits. One such fitness-correlated trait is drought tolerance, a selection target that varies among environments with different precipitation conditions [23]. In drought conditions, water loss is highly detrimental to fitness, so drought tolerance should be strongly favoured [24]. As well, crop plants are thought to have better drought tolerance than their wild relatives [25, 26], so altered precipitation patterns by human-mediated global climate change may have a substantial effect on selection between wild and hybrid weeds [27, 28]. In agricultural plant communities, where crop and compatible weedy relatives coexist, we may expect different life-history responses to identical environmental cues [29]. Contrasting strategies of resource allocation in high- and low-stress environments may be driven by adaptive physiological and morphological differences between wild and crop plants [30, 31]. For example, drought-adapted plants can minimize water loss in drought conditions by closing their stomata, though this will convert fewer carbon resources [24]. If crop plants hybridize with wild relatives, the resulting crop-wild hybrid offspring could exhibit a combination of alternate life-history strategies, and potentially succeed in environments that exclude or minimize the weediness of the wild parent [32].

Geographic and temporally shifting climatic conditions can affect plant phenology and fecundity, key fitness-related traits [33]. In particular, variation in precipitation patterns may have dramatic consequences for the subsequent fitness of crop-wild hybrids. Climate models predict punctuated and extreme rainfall over terrestrial regions, including more severe drought and flooding events [34]. Changing climate may impact agricultural ecosystems in particular, where farmers rely on high rates of germination and survival, and high fecundities in cultivated species while attempting to minimize these same life-history traits in weedy competitors.

Ecological risk-assessments of weeds derived from crop-to-wild hybridization have largely based their conclusions on one or a few stages of a plant’s life cycle (e.g., female fecundity), which may not accurately predict population dynamics [35]. In fact, assessing seed production alone would have supported incorrect conclusions relative to complete life-history data in crop-wild systems of *Lactuca* and *Raphanus*, or above- and below-ground biomass of *Brasica rapa* and *B. napus* hybrids [2, 13, 36]. If crop-wild hybridization alters weed life history [e.g., [9]], these new life histories may affect vital rate contributions to population growth and alter the effectiveness of weed management practices [36]. In summary, there is little information that projects the impact of crop-wild hybridization events combined with extreme rainfall or drought events on the life-history dynamics of invasive weed populations.

To assess the impact of crop-wild hybridization and projected environmental variation (specifically water availability) on the population dynamics of nascent crop-wild hybrid populations, we used a Life Table Response Experiment (LTRE). A LTRE decomposes a cumulative dependent variable into its contributing metrics that compose that dependent variable. The population dynamics of weedy phenotypes are dictated by multiple interacting components of fitness that may be best captured by a demographic modeling approach. Transitions among key life-history stages (e.g., rates of germination, survival and reproduction) are closely associated with individual fitness and collectively contribute to population growth, often measured as an asymptotic rate of population growth (λ) [37]. For example, fecundity strongly contributes to *λ* in the crop-wild *Raphanus* complex [4, 38]. A LTRE tests for the consequences of experimental manipulations on a population’s growth, using demographic transitions (or vital rates) as well as assessing the sensitivity of the model to proportional conversions between stages [39, 40]. Because sensitivities reflect a functional correlation with multiple components of fitness, they can be interpreted as the strength of selection acting on a particular life-history trait [37, 41].

A central challenge in research of annual, agricultural weeds is to identify how their success can be curbed by control measures. Weeds may be considered difficult to control or invasive when they exhibit rapid population growth, longer population persistence (due to denser populations, seed banks or aggressive competitive abilities), and a greater ability to disperse or found new populations (framework described in [38]). When selection is applied to biological populations, we expect population size to decline and a lowered probability of long-term persistence [42–44]. Alternatively, although strong selection may reduce population size, the resulting population may be at a higher fitness peak and thus should persist for a longer period of time [45, 46]. Here, we focus on assessing the relationship between natural selection on wild versus hybrid genotypes grown under a range of moisture gradients and their problematic persistence.

To test the prediction that segregating crop-wild hybrid populations will experience stronger selection and hence reduced population size, relative to non-hybrid weed populations, we compared the population growth rates and the strength of selection on vital rates of wild versus crop-wild hybrid *Raphanus* populations across an experimental moisture gradient [37, 47, 48]. Through our experimental demography approach, we explore how selection on weedy life histories can be altered by moisture gradients and anticipate weed evolution in variable climates.

## Methods

### Study species

To explore the role of hybridization and soil moisture application in weed invasion, we used the *Raphanus* (Brassicaceae) crop-wild complex. Here, the term ‘biotype’ subsequently refers to the taxa: wild *R. raphanistrum* biotype, crop *R. sativus* biotype, and the hybrid *R. raphanistrum* x *R. sativus* biotypes. All three biotypes are self-incompatible and insect-pollinated [49]. Crop and wild *Raphanus* biotypes can be distinguished by petal colour, a simply inherited Mendelian trait, where the white-petal allele (a crop trait seen in hybrids) is dominant and the yellow-petal allele (a wild trait) is recessive [50]. Therefore, F_1_ crop-wild hybrids are 100% white flowered and advanced generations often exhibit mixtures of white and yellow flowered plants, depending upon the hybridization rate [3].

These annual plants have a sequential life cycle, subject to mortality at any stage. Further, *R. raphanistrum*, with a long-lived seed bank, high genetic variability, and early emergence after soil disturbance, is considered a weed in more than 45 crop systems and 65 countries [51], so changes to its spread and persistence could impact a large diversity of agricultural cropping systems. The wild biotype, *R. raphanistrum* exhibits delayed germination with a long seasonal range but reaches reproductive maturity earlier than the crop biotype, *R. sativus,* which germinates synchronously within a few days of planting and flowers late in the season [9, 49]. These differences in key life-history traits may have important implications for the weediness of their hybrid offspring. Hybrid biotypes can benefit from inheriting germination and flowering times from crop biotypes within agricultural environments.

### Study site

We conducted this study at the Koffler Scientific Reserve (KSR) at Joker’s Hill, King City, ON Canada (44°0’ N; 79°3’ W, 285 m asl) for field experiments in abandoned agricultural fields from 2011-2013, involving F_1_ to F_3_ generation offspring. Here, the growing seasons during which we conducted our field experiments spanned the period of May 24th - October 25th.

### Establishment of replicated populations

During the summer of 2010, 36 populations of nine F_0_ cultivated and nine F_0_ wild plants were grown in field plots in Columbus, OH and exposed to one of four watering treatments (no rain, double rain and two types of control plots), as described in [52] to create wild and hybrid offspring at naturally occurring rates. To determine if crop-wild hybrid radish populations experience stronger selection on demographic traits than wild radish, in 2011, we established 24 replicated populations (plots) of wild or F_1_ crop-wild hybrid radish seeds. First generation (F_1_) seeds were collected from F_0_ wild radish that had either mated with F_0_ wild radish or had mated with F_0_ crop radish, respectively. Seeds were collected only from wild radish mothers for experimental use as the wild or crop-wild hybrid F_1_ generation in 2011 to allow us to accurately describe the biotype of offspring. Plots were separated by > 40 m to prevent gene flow. Due to environmental variation across KSR, we used a randomized complete block design with three blocks, each including one plot per watering treatment per biotype. Plots were tilled by May 15th, annually. These experimental populations have been previously described [53].

Two weeks after germinating in a greenhouse, 117 randomly selected F_1_ seedlings were transplanted into plots, 15 cm apart. As plants flowered, petal colour was noted; wild plants possessed yellow petals and hybrid plants possessed white petals. Plots were randomly assigned a biotype treatment; any plant representing the “wrong” biotype relative to the assigned biotype was removed upon detection. This created the opportunity for a very small amount of gene flow to occur (detected in one “wild” plot in 2012). Flowering plants fruited and senesced naturally, producing F_2_ in 2012 and G_3_ in 2013 (individuals in G_3_ could have been F_2_ or F_3_ genotypes, due to seed dormancy). Annually, seeds from a representative group of plants (maximum 30 individuals) were collected to assess individual fecundity; otherwise seeds dispersed naturally.

### Experimental treatments

To manipulate soil moisture, we imposed four watering treatments from July 1–August 31, 2011, June 4 – August 31, 2012 and June 10 – September 6, 2013. Rainout shelter roofs were made from translucent plastic attached to 7.44 m^2^ wooden frames elevated 1.2 m above ground and slightly sloped northwest. Intercepted precipitation was collected in rain barrels and we applied watering treatments within two days of collection. Only 15 of 24 plots were used to measure *λ* due to low seed production in 2011 (replication described below). We hand weeded plots to minimize interspecific competition.

We administered the following four watering treatments: 1) Control Unsheltered (CU), where rainwater fell naturally on plots (analyses of *r*: 3 wild, 3 hybrid plots); 2) Control Shelter (CS), where plots assessed effects of rainout shelters, and collected rainwater was reapplied to the plot (analyses of *r*: 3 wild, three hybrid plots; analyses of $\lambda$: 3 wild, 2 hybrid plots); 3) No Rain (LR), where plots assessed effects of reduced precipitation, so collected rainwater was withheld (analyses of *r*: 3 wild, 3 hybrid plots; analyses of $\lambda$: 1 wild, 3 hybrid plots); 4) Double Rain (DR), where plots assessed effects of increased precipitation, thus DR plots received the normal rainfall plus rainwater collected from LR plots within the same block (analyses of *r*: 3 wild, 3 hybrid plots; analyses of $\lambda$: 3 wild, 3 hybrid plots). The LR and DR treatments were designed to simulate extreme precipitation patterns that are projected to result from climate change [34]. Experimental watering treatments significantly and predictably altered the average volumetric moisture content in both years [53]. Control sheltered plots had significantly lower soil moisture than double rain plots and significantly higher soil moisture than no rain plots.

### Annual surveys of replicated populations

Whereas $\lambda$ describes the proportional change in population size in discrete time, the exponential growth of weed populations may also be described by the intrinsic rate of increase, *r*. To estimate *r*, annual counts of flowering and non-flowering individuals were conducted in 2012 and 2013. Using abundance estimates of each whole plot in 2011 and within subplots (see subplot details below) in 2012 and 2013, we calculated plant density (number of individuals/cm^2^) to determine how population growth rate (*r*) changed through time. Instantaneous population growth rate (*r*) was calculated as the difference in natural log transformed population size density (*N*) for year_*t*_ and year_*t-1*_. We ran a mixed-model ANOVA to determine whether biotype, watering treatment, or their interaction or block resulted in significant changes in *r*.

### Weekly surveys of replicated populations

Annually, we established a 1 m^2^ subplot in the centre of each plot, within 16 days of tilling. We intended to follow ~50 plants per subplot, but plant density varied between subplots. When more than 50 plants germinated within a subplot, we methodically reduced subplot size until it contained ~50 plants.

Weekly censuses monitored four key life-history stages of seed, cotyledon, non-flowering adult and flowering adult that described five vital rates of seed germination, seed dormancy, survival-to-non-flowering adult, survival-to-flowering adult and fecundity (as in [38, 53]). Seed dormancy was estimated from results of a companion seed burial experiment [53], such that dormancy vital rates were weighted by dormancy rates of their corresponding plot and removal date, adjusted for population size. Mortality could occur at each stage and is reflected in the proportion of individuals that survive to the next life-history stage. A plant that went unrecorded for > two weeks was presumed dead. If a plant was rediscovered, and the plant either did or did not mature from the last known entry, we filled in the missing week(s) with the last known status. Data are recorded in [54].

Plants were harvested as they senesced or at the last weekly census, after the first frost when there was no new fruit development (Z. Teitel, pers. obs.). Above-ground biomass was collected in paper bags and dried in an oven for *ca*. seven days at 30 °C. For 30 randomly selected, reproductive plants per sub-plot (or all plants when population size ≤ 30 reproductive plants), we counted the number of fruits per plant. Number of seeds per fruit was assessed by counting locules in a silique for 10 randomly chosen fruits per plant. To estimate the number of seeds per plant, we multiplied the average number of seeds per fruit by the number of fruits.

### Analysis of population demography

We used a fixed-effect life table response experiment (LTRE; [37]) to compare lambda (*λ*) of each experimental population each year (48 constructed matrices; e.g., wild double-rain replicate 1, hybrid no-rain replicate 2, etc.) as a linear function of biotype (*g*), watering treatment (*w*), and their interaction (*gw*): − *λ* ^*gw*^ = *λ* ^(⋅⋅)^ + *α* ^*g*^ + *β* ^*w*^ + *αβ* ^*gw*^ where *α* ^*g*^ is the effect of the *g* ^th^ level of the biotype, β^*w*^ is the effect of the *w* ^th^ level of the watering treatment, and *αβ* ^*gw*^ is the interaction of the *g* ^th^ biotype and *w* ^th^ watering treatment, measured relative to the projected growth rate of a reference matrix ^(..)^.

To obtain the treatment matrices, we first averaged all replicates of matrices belonging to a given treatment combination (e.g., the transition frequencies of double-rain replicates 1, 2, and 3 for wild and hybrid populations were averaged) [4, 55]. We then averaged common treatment groups of these matrices to give us mean representative matrices for a given treatment (mean wild type, mean double-rain, etc.). We estimated treatment effects as:

$$ {\alpha}^g={\lambda}^{g.}-{\lambda}^{..}\cdot \cdot \approx \kern0.5em \sum \left.\left[{\alpha}_{ij}^g-{\alpha}_{ij}^{\cdot \cdot}\right]\kern0.5em \left(\delta \lambda /\delta {\alpha}_{ij}\right)\right|\left[{A}^{g.}+{A}^{\cdot \cdot}\right]/2 $$

$$ {\beta}^w\kern0.5em =\kern0.5em {\lambda}^{\cdot w}-{\lambda}^{\cdot \cdot}\cdot \approx \sum \left.\left[{\alpha}_{ij}^{\cdot w}-{\alpha}_{ij}^{\cdot \cdot}\right]\cdot \left(\delta \lambda /\delta {\alpha}_{ij}\right)\right|\left[{A}^{\cdot w}+{A}^{\cdot \cdot}\right]/2 $$

$$ \alpha {\beta}^{gw}={\lambda}^{gw}-{\lambda}^{\cdot \cdot }-{\alpha}^g-{\beta}^w\cdot \approx \sum \left.\left[{\alpha}_{ij}^{gw}-{\alpha}_{ij}^{\cdot \cdot}\right]\cdot \left(\delta \lambda /\delta {\alpha}_{ij}\right)\right|\frac{\left[{A}^{gw}+{A}^{\cdot \cdot}\right]}{2}-{\alpha}^g-{\beta}^w $$

where we obtained elasticities (as described in [42]) and sensitivities (*δλ/δa*_*ij*_) from the relationship *δλ/δa*_*ij*_ = *v*_*i*_ *w*_*j*_/<*w,v* > where *a*_*ij*_ is a matrix element in the *i* ^*th*^ row and *j* ^*th*^ column, and *v* and *w* are the right and left eigenvectors of the matrix and evaluated the sensitivities, halfway between the reference and treatment matrices [37]. Sensitivities describe how *λ* changes in response to changes in matrix elements, and elasticies are standardized sensitivities. We obtained treatment matrices (e.g., A^g.^, A^.w^) by pooling data across all levels of the other treatments. Finally, the contributions were calculated by weighting the differences in vital rates by their sensitivities. Therefore, the above equations describe both observed variation in matrix elements and the sensitivity of population growth to variation in those elements that influence the effect of treatments on population growth. A particular matrix element *a*_*ij*_ may contribute little to variation in *λ* in cases when *a*_*ij*_ was invariant among treatment classes and/or when *λ* was insensitive to variation in *a*_*ij*_. Additionally, *a*_*ij*_ may contribute to little variation in *λ* even if *λ* was highly sensitive to the element if the vital rate did not differ among treatments. In alternate scenarios, even small amounts of variation in *a*_*ij*_ may drive variation in *λ* when there are consistent differences among treatments and when *λ* is highly sensitive to that matrix element. Matrix algebra and analyses were performed using MATLAB (v.2012a; The Mathworks, Inc., Natick, Massachusetts, U.S.A).

To determine how biotype and environment contribute to *λ*, we conducted a Type III ANOVA in which biotype, watering treatment, block, and their interaction were fixed effects for the response variable of *λ*. We analyzed each year separately because the model did not have enough degrees of freedom for this factor to be included. To determine how biotype and environment affect the sensitivity of *λ* to vital rates (i.e., the selection gradient), we conducted a repeated measures ANOVA in which biotype and watering treatment were between-subject effects, and year, biotype and watering treatment were within-subject effects. In both models, block was not a significant effect in the model and was removed. We used SPSS Statistics 21 (2012; SPSS Inc., Chicago Illinois USA) for all analyses.

## Results

### The strength of selection on weed vital rates

**Fig. 1:**
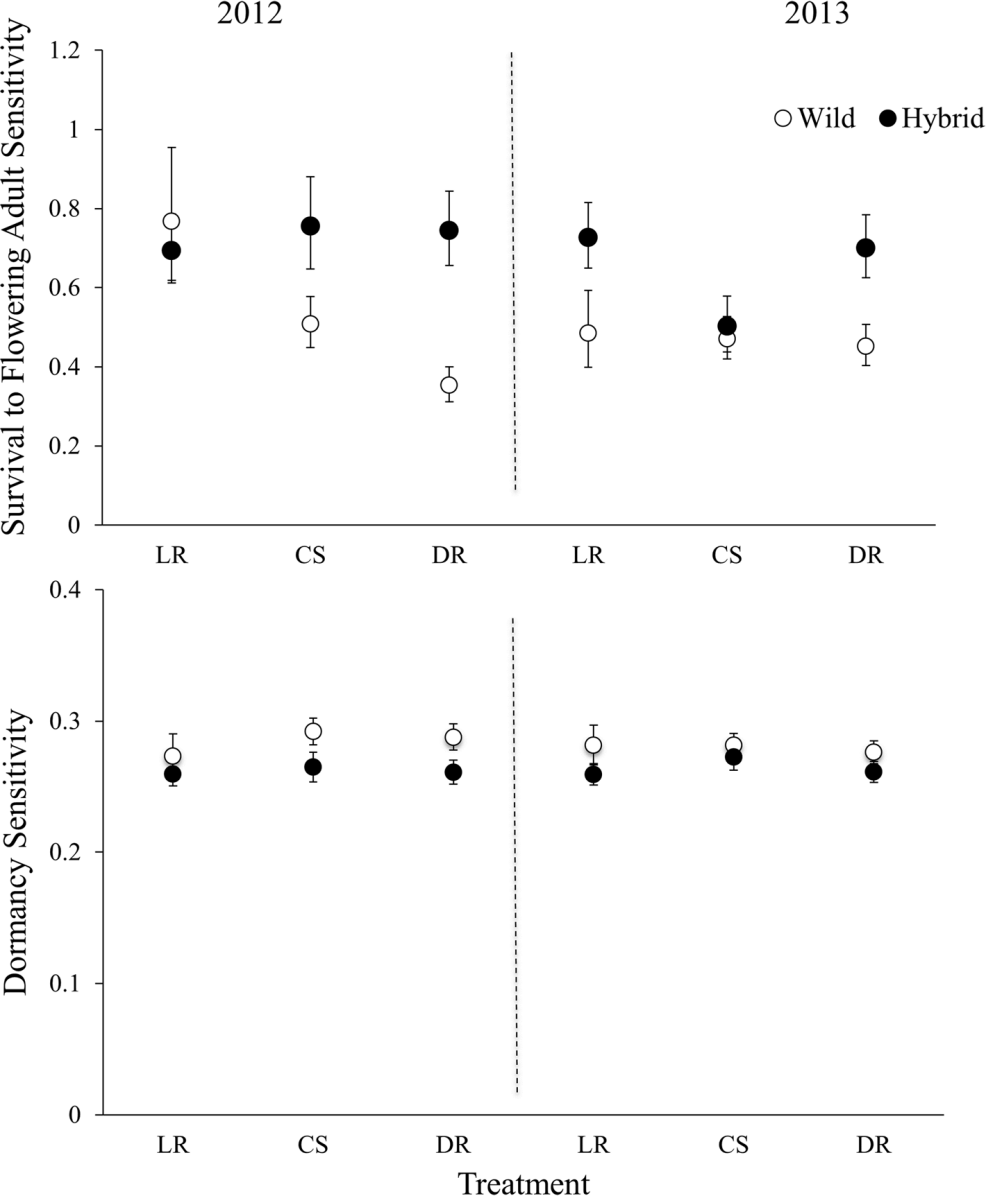
*Top*: Back-transformed sensitivities of survival-to-flowering adult, or *Bottom*: Back-transformed sensitivities of dormancy, between hybrid and wild populations of *Raphanus*, under three watering treatments [low rain (LR), control shelter (CS), double rain (DR)], grown in King City, ON, in 2012 and 2013. ‘Sensitivity’ from a vital rate is calculated as the absolute change in *λ* resulting from a change in one of the vital rates

Selection, measured as the sensitivity of *λ* to proportional changes in vital rates, varied significantly with year, soil moisture, and biotype for at least one vital rate (Table 1). Therefore, we explored the effect of soil moisture and biotype for each year independently (Table 1b, c). Selection for survival-to-flowering adult was about 8.7 times stronger on hybrid relative to wild biotypes in 2012 (Table 1b, Fig. 1) and 10.3 times stronger in 2013 (Table 1c, Fig. 1a). Selection for dormancy was ~10% stronger on wild relative to hybrid biotypes, in 2012 (Table 1b) and only marginally stronger in 2013 (Table 1c, Fig. 1b). The strength of fecundity selection differed significantly among years. It is interesting to note that rainfall differed between 2012 and 2013, during the period when *Raphanus* seeds most frequently germinate (May), such that control plots received twice as much rainfall during that period in 2013 as 2012 (data from KSR research station, summarized in [53]). The strength of selection for survival-to-flowering adult and dormancy did not differ significantly across soil moisture treatments (Table 1b and c, Fig. 1). Furthermore, the strength of selection on emergence, survival-to-vegetative rosette or seed production did not differ significantly across biotype or watering treatment in both years (Table 1 b and c).

**Table 1:** Repeated measures ANOVA (a) and two-way ANOVA in 2012 (b) and 2013 (c) for biotype, watering treatment and their interaction on strength of selection as measured by sensitivity of *λ* to proportional changes in dormancy, emergence, survival-to-non-flowering adult, survival-to-flowering adult, and fecundity

Source of Variation	Dormancy	Emergence	Survival to Non-flowering Adult	Survival to Flowering Adult	Fecundity
*(a) Repeated measures ANOVA:*					
Between subject effects:					
biotype	5.622_1,9_*	6.245_1,9_*	1.556_1,9_	9.591_1,9_*	0.018_1,9_
watering treatment	0.461_2,9_	0.109_2,9_	0.831_2,9_	1.289_2,9_	0.252_2,9_
biotype*watering treatment	.018_2,9_	0.26_2,9_	0.922_2,9_	1.901_2,9_	0.879_2,9_
Within Subject Effects:					
year	.049_1,9_	5.067_1,9_^+^	13.836_1,9_**	5.061_1,9_^+^	15.955_1,9_**
year*biotype	.893_1,9_	0.526_1,9_	0.8_1,9_	0.176_1,9_	0.092_1,9_
year*watering treatment	0.556_2,9_	1.833_2,9_	3.379_2,9_^+^	5.064_2,9_*	0.308_2,9_
year*biotype*watering treatment	0.94_2,9_	0.007_2,9_	2.986_2,9_	5.726_2,9_*	1.571_2,9_
*(b) 2012*					
biotype	5.806_1,9_*	4.519_1,9_^+^	1.755_1,9_	7.979_1,9_*	0.052_1.9_
watering treatment	0.475_2,9_	0.839_2,9_	1.666_2,9_	2.788_2,9_	0.131_2,9_
biotype*watering treatment	0.17_2,9_	0.097_2,9_	1.541_2,9_	3.796_2,9_	1.114_2,9_
*(c) 2013*					
biotype	3.543_1,9_^+^	3.406_1,9_^+^	1.022_1,9_	7.388_1,9_*	0.000_1,9_
watering treatment	0.482_2,9_	.573_2,9_	0.202_2,9_	1.137_2,9_	0.455_2,9_
biotype*watering treatment	0.185_2,9_	.295_2,9_	0.505_2,9_	1.291_2,9_	0.839_2,9_

^+^indicates *p* < 0.1, *indicates *p* < 0.05, and **indicates *p* < 0.01. Data was natural logarithm transformed; sphericity was assumed

### Population growth rates of wild and crop-wild hybrid populations across a moisture gradient

**Fig. 2:**
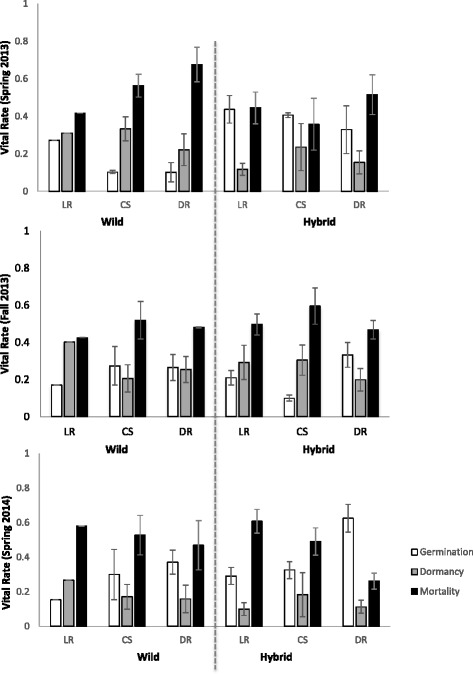
Comparison of average instantaneous population growth rates (*r*) of wild and crop-wild hybrid populations of *Raphanus* grown under four watering treatments [low rain (LR), control unsheltered (CU), control shelter (CS), double rain (DR)] in King City, ON (+/- SE) from 2011-2013

**Fig. 3:**
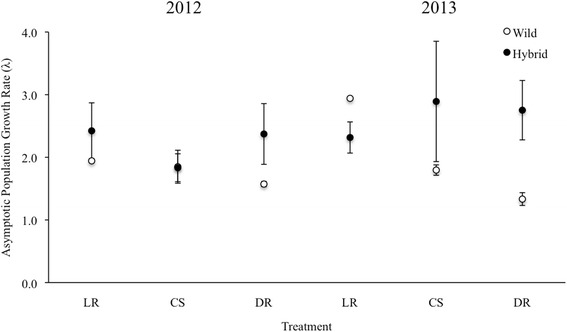
Comparison of average asymptotic population growth rates (*λ*) of wild and crop-wild hybrid populations of *Raphanus* grown under various watering treatments [low rain (LR), control shelter (CS), double rain (DR)] in King City, ON (+/- SE) for 2012-2013. Comparisons were made within years not between years

From F_1_ to F_3_, hybrid populations had higher instantaneous population growth rates (*r*) than wild populations (Fig. 2; *F*_1_,_14_ = 37.18, *P* < 0.001). In the first generation of population growth (F_1_-F_2_), hybrid populations grew four times faster than wild populations (Fig. 2; *F*_1,14_ = 29.43, *P* < 0.0001). In the following generation, we saw no significant difference in population growth between biotypes (Fig. 2; *F*_1,14_ = 1.17, *P* = 0.30). Hybrid populations also had marginally significantly higher asymptotic population growth rates, when measured as *λ*, across years (Fig. 3).

Across two years, soil moisture did not significantly affect *r* (Fig. 2; F_3,14_ = 1.52, *P* = 0.25). However, in the first year, LR populations grew slower than populations experiencing other soil moisture treatments (Fig. 2; F_3,14_ = 6.35, *P* = 0.0061; X_LR_ = 0.3731, X_CU_ = 2.8956, X_CS_ = 3.0633, X_DR_ = 3.9647). In contrast, in the second generation, LR populations exhibited significantly higher *r* than populations grown in other soil moisture treatments (Fig. 2; F_3,14_ = 3.38, P = 0.048; X_LR_ = 2.0522, X_CU_ = -0.4936, X_CS_ = 0.4472, X_DR_ = 0.04061). In the first generation, wild populations grown in LR conditions had a significantly lower *r* than any other biotype-by-environment combination (Fig. 2; F_3,14_ = 3.94, *P* = 0.031). However, we saw no significant biotype-by-environment interaction across both generations (Fig. 2; F_3,14_ = 1.25, *P* = 0.33), or in the second generation (Fig. 2; F_3,14_ = 1.37, *p* = 0.29). In contrast to the response of *r* to experimental manipulation, neither watering treatment, nor its interaction with biotype had a significant effect on population growth rate measured as *λ*, across years (2012, 2013) (Fig. 3).

### The effect of vital rates on population growth across an environmental gradient

**Fig. 4:**
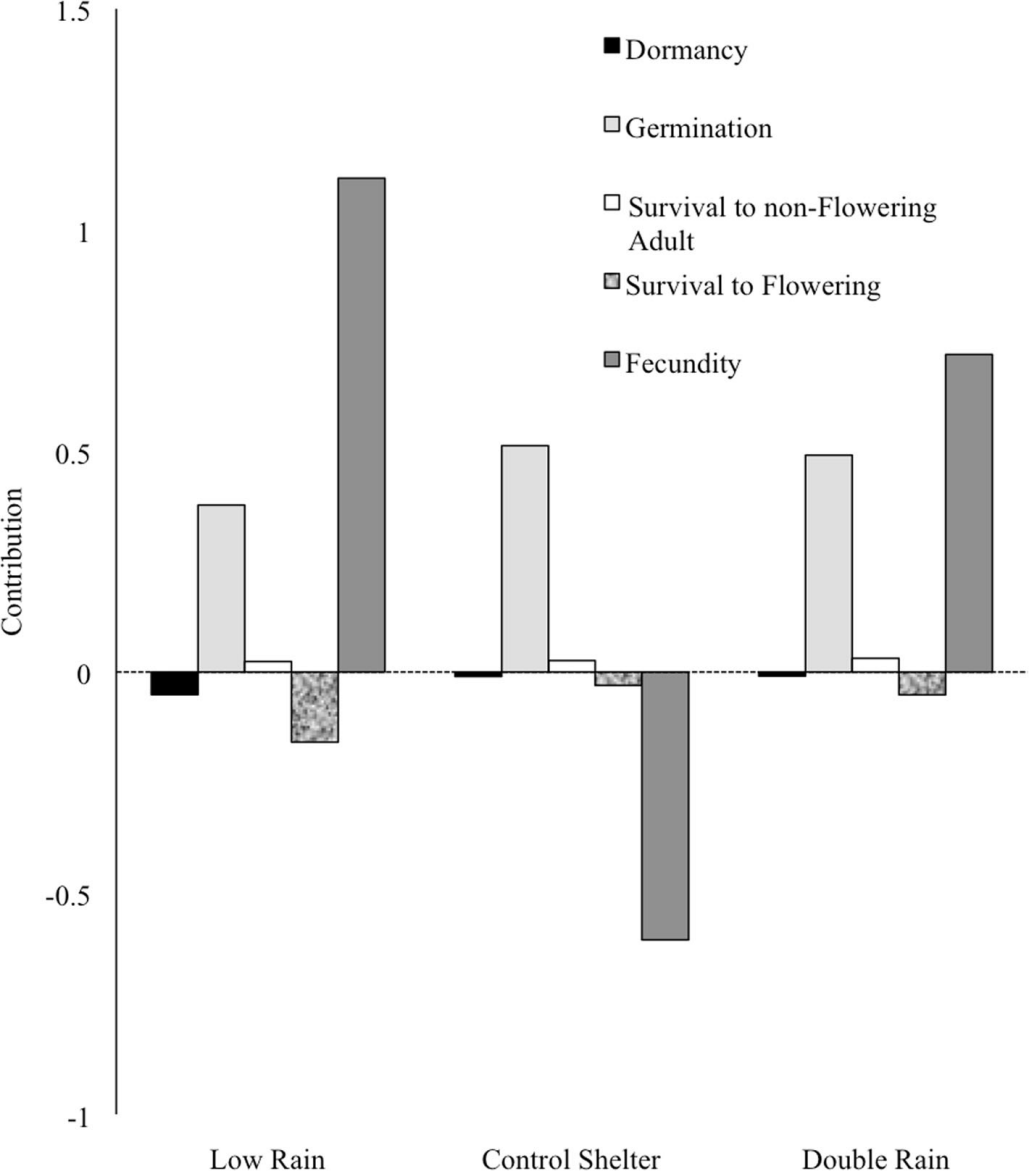
Contributions from dormancy, germination, survival-to-non-flowering adult, survival-to-flowering, and fecundity vital rates of *Raphanus*, to differences between hybrid and wild biotype population growth rates (Δ *λ*), under three watering treatments [control shelter (CS), double rain (DR), low rain (LR)], grown in King City, ON, from 2012-2013. A ‘contribution’ from a vital rate is calculated as the ‘difference’ in corresponding matrix elements (wild – hybrid), weighted by its ‘sensitivity’ to describe how lambda changes with different vital rates

Life-history transitions contributed to changes between hybrid and wild average population growth rates to different degrees and directions (Fig. 4). In general, fecundity contributed most to differences in $\lambda$, followed by germination, then survival-to-flowering adult and finally survival-to-non-flowering adult and seed dormancy (Fig. 4). Differences in germination and survival-to-non-flowering adult between hybrid and wild populations led to higher relative population growth rates in hybrid versus wild populations (Fig. 4). In contrast, differences in dormancy and survival-to-flowering adult led to higher relative population growth rates in wild versus hybrid populations (Fig. 4). Finally, fecundity only contributed positively to hybrid $\lambda$ relative to wild $\lambda$ when precipitation was altered and not under the CS treatment (Fig. 4).

## Discussion

Our results contribute to the relatively small body of work that investigates natural selection in field populations of weedy and invasive plants (e.g., [9, 56–58]) and the growing number of studies that use demographic sensitivities to estimate the direction and strength of selection therein [41, 59]. When a weed colonizes a new location, it may often find itself in a novel environment with selection pressures that are different from its source population. The ability to respond rapidly to these novel selection pressures is perhaps one method in which plants may evolve traits that promote increased weediness (i.e., rapid population growth, longer population persistence, and/or greater ability to found new populations) [60]. Here we documented selection acting on multiple life-history stages of wild and crop-wild hybrid *Raphanus* ssp. Selection analyses revealed that selection strongly favoured both wild and hybrid phenotypes that survived to reproduction in both survey years. In contrast, wild populations experienced stronger selection for dormancy than hybrid populations in 2012 but not 2013, and this difference in selection is perhaps due to differences in water availability, albeit non-experimentally controlled. Further, selection for juvenile survival to the rosette stage was marginally stronger on hybrid than wild populations and selection was negatively related to soil moisture. Dry spring conditions in 2012 resulted in relatively less population growth (measured as *r*) in wild populations only, in direct contrast to the extremely positive growth exhibited by all of other biotype-by-watering treatment combinations. Moreover, fecundity selection acted equally strongly on both biotypes and across watering treatments but was significantly stronger in 2012 relative to 2013. Finally, the differences in population growth among biotypes and watering environments were most influenced by seed production, rather than other life-history stages. Yet, fecundity only contributed positively to higher hybrid population invasiveness relative to wild populations when precipitation was altered and not under the control shelter treatment. These results suggest that weed populations, although predictably most influenced by fecundity selection, may differ in selective environments based on genotype (including the strength and direction of selection on crop-derived traits), or environmental context.

### Selection on weeds

Although we detected only marginally significant differences in *λ* between biotypes, wild and hybrid populations attained similar population growth using alternate life-history strategies. Rates of germination and survival-to-rosette boosted *λ* of hybrid populations, whereas rates of dormancy and survival-to-flowering boosted *λ* of wild populations. These results satisfy our prediction that crop seed banks are expected to have synchronous and heightened rates of germination, as well as minimal dormancy, due to continuous selection for domesticated traits [61]. In contrast, wild populations may be subject to a wider range of environmental conditions and can respond to unfavorable growth conditions with prolonged dormancy or staggered emergence [62]. However, the literature surrounding the escape of domesticated, functional traits into weed populations often predicts the opposite result, i.e., that such crop-derived trait are generally expected to be purged from weed populations (e.g., [35, 49]). Selection for an optimal hybrid life-history could result in tradeoffs with other traits. Though data is scarce, there is some evidence that annual weed species suffer from post-emergence mortality in particular [63]. When compared to planted crops, they have far fewer seed reserves to rely on during emergence and seedling establishment [64]. Thus, the relative importance of seed dormancy function is crucial for weed propagation strategy.

Demographic studies that conduct sensitivity analyses, such as ours, often focus on the ecological outcomes and less on the evolutionary interpretations of their findings, making it difficult to place the magnitude of our estimates of selection into context. Conversely, few empirical estimates of strength of selection have been achieved by conducting a life table response experiment [65], and applying sensitivity analyses to measure selection on individual vital rates that describe the entire life history of an organism; those that have used this approach tend to address basic biological questions (e.g., [41, 66, 67]). This is despite the considerable value that such prospective analyses have in projecting the spread of invasive weeds [68, 69] and predicting how strongly and in what direction *λ* will respond to changes in vital rates due to broad ecological effects [70]. In an earlier comparison of selection on morphological traits in wild and hybrid radish, selection was strongest on number of flowers, a correlate for fecundity selection [9], relative to other morphological traits (and survival was nearly 100% due to experimental watering). The strength of selection, measured here using sensitivity analysis, appears to be stronger on estimates of demographic transitions than estimates of selection on morphological traits, based on a Lande-Arnold regression approach and short-term correlates of fitness (i.e., total, individual seed production [9]). Though our study supports the conclusion that selection acting on survival may be greater than that on fecundity, the vast literature of published plant demographic matrices [71] has yet to present any conclusive analyses on selection for comparison.

### Is Weediness Environmentally Dependent?

Both biotic and abiotic interactions can alter weedy radish population growth; however, we found limited evidence that soil moisture manipulations dramatically altered long-term population growth of these weeds. Instead, drought conditions resulted in shrinking population sizes (measured as *r*) in wild populations in 2012 only, in direct contrast to the positive growth exhibited by all other biotype-by-watering treatment combinations. Perhaps more intriguingly, we have documented that weeds alter life-history strategies to accomplish the same rate of growth, depending on the environment. For instance, fecundity contributed positively to higher hybrid population invasiveness relative to wild populations only when precipitation was altered and not under the control shelter treatment. This work, where only soil moisture varied, supports previous work that has documented the context dependency of hybrid genotypes across North America, where many environmental variables differed among locations [3, 72]. However, we may have seen differences in *λ* among treatments if there been a larger seed-bank sample size [55] or if the watering treatments were extended to winter months.

Consistent with our results, the literature shows numerous examples of plant demographic parameters sensitively responding to changes in precipitation (e.g., [73, 74]). Whereas drought conditions can cause extensive mortality in weed species over crops, our results did not reveal any effects of the environment or the biotype-by-environment interaction on *λ*. This wide environmental tolerance suggests *Raphanus* spp. can plastically respond to the range of altered moisture conditions we created. Successful plant invaders often need to employ phenotypic plasticity for a wide range of extreme environments [75] and *Raphanus* appears well to be prepared for both drought and excess moisture conditions.

### Demographic analysis – an under-appreciated risk assessment tool

Ultimately, studies of crop-wild hybridization assess whether crop-derived traits will persist in wild or weedy populations [1, 76]. One significant shortcoming of fecundity studies to address this question is the potential for low correlation between the number of seeds produced and fitness ([77]; although this is more common in perennial plants; [78]). In contrast, demographic estimates can account for both multi-generational survival and reproduction [37]. Certainly, in our study, although fecundity was the largest influence on population growth, differences in survival also played an important role in the differences in growth rate of these new populations and some risk assessment literature advocates their use [2, 79]. Here, we propose a second way they can be useful as a predictive, risk assessment tool. Selection estimates derived from sensitivity analyses can clearly be used to predict whether crop-derived demographic traits (e.g., loss of dormancy, proportion of the population that transition from vegetative rosette to flowering, given that flowering is delayed in crop populations) will persist within weed populations. Based on our results, we predict that selection will favor loss of dormancy in crop-wild hybrid populations (i.e., selection for the crop-derived trait) and the delayed flowering that reduces the proportion of plants that survive to flower (i.e., selection against the crop-derived trait). Perhaps future demographic studies could modify the frequency of these traits within experimental populations to test whether the populations persist longer and grow faster (e.g., [4]). Alternatively, individual-based models, which characterize genotypes into groups of demographic strategies [80], could be used to compare the consequences of demographic trait variation in frequencies of possessing crop and wild traits.

## Conclusion

Our goal was to develop a quantitative understanding of the selection pressures experienced by weedy *Raphanus* and to ultimately develop predictions about the direction and pace of evolution under changing moisture conditions. Although field studies of selection in genetically diverse experimental populations growing under natural and manipulated conditions are transforming our understanding of selection dynamics [81, 82], few studies have employed a cumulative estimate of fitness and sensitivity analysis to estimate hybrid population growth [e.g., 59] or have addressed the specific issue of selection in weedy or invasive populations [9, 56–58]. This work reveals the context-dependency of the selective advantage of domesticated traits to the long-term population dynamics of weedy *Raphanus* populations.
